# Notes of Some Cases of Injury Recently Treated in the “Emergency Hospital”

**Published:** 1885-11

**Authors:** Herbert H. Mickle


### Clinical Notes.

### Notes of some Cases of Injury Recently Treated in the "Emergency Hospital." [Compd. fractures]

Reported by Herbert Mickle, M. D., M. R. C. S.

W. L., æt. 17, was admitted to the Emergency Hospital August 19th, with a railroad crush of right foot and lower two-thirds of right leg. The terminal phalanges and soft parts of first and second toes of left foot were also found crushed. This accident took place 20 miles out of town. The pulse, on admission, was 130 to the minute, with well-marked symptoms of shock. Amputation at upper third of the leg, oval-flap method, with periosteal flap; the bone divided just below insertion of ligamentum patellæ.

Dressings were changed on the fourth day, and again on the tenth. Patient discharged on the sixteenth day with a sound and painless stump, healing having taken place without any visible suppuration.

J. C., æt. 22, a farmer, was admitted September 1st with a railroad crush of right foot and lower third of right leg. Amputation through middle of calf of right leg by oval-flap method, with periosteal flap. Temperature, 48 hours after operation, was 100° F.; after that time it was not found above 99.2° F. The dressings were renewed on the third day, and again on the eighth, when drainage tube was removed. No pus was noticed during healing, and on the fifteenth day this patient was discharged with a sound and perfectly painless stump and in good general condition.

R. R., æt. 34, a farmer, was admitted September 19th with a railroad crush of right leg as far up as the knee joint. Amputation at lower third of the thigh by oval-flap method, periosteal flap; femur divided at junction of its middle and lower third. Patient rallied well from a condition of profound shock. Forty-eight hours after operation, temperature was 101° F., and this was the highest point noted. Dressed on the sixth day, when union of the flaps appeared complete, and drainage tube was removed; dressed again on the tenth day. On the seventeenth day this patient was discharged in good health, and with a perfectly sound stump, free from pain and tenderness.

In these cases, antiseptic precautions, as practiced regularly in the Hospital, were carried out strictly. These embrace a preliminary shaving of the limb to be operated on, followed by careful sponging with the turpentine and spirits mixture; the use of catgut ligatures with bichloridised silk sutures; the careful arrest of all hemorrhage, including oozing; thorough irrigation with mercuric bichloride solution of a strength of 1 in 2000; boracic acid, in impalpable powder, freely dusted on the stump, and a dressing of wood wool, impregnated with bichloride of mercury and napthaline, and put up in bags of coarse cheese-cloth of various sizes; the use of the rubber drainage tube, which in each case was removed early to avoid any irritating influence. The sponges used are renewed often and kept in a strong solution of carbolic acid. The instruments are laid out in a solution of carbolic acid in water, 1 to 30. The operator's and assistant's hands washed in a solution of corrosive sublimate, 1 part to 2000 of water.

J. L., æt. 35, admitted August 14th. This man fell from a ladder, a distance of about ten feet, and was brought to the Hospital with a compound fracture of left leg, tibia and fibula, about junction of upper and middle third. There was a wound of soft parts, three inches long, on anterior aspect of leg at that point, through which the upper fractured end of tibia and some lacerated muscle protruded. This wound was irrigated with bichloride solution, a "posterior plaster splint" applied to the limb, leaving an opening corresponding to the injury of soft parts on the anterior aspect, over which an antiseptic dressing of wood wool was used, as in the cases of amputation related. The temperature on the evening of the first day was recorded as 103° F., and from this time declined to 99° F. on the seventh day, with a slight evening rise. On the twentieth day, this patient left the Hospital for reasons unconnected with the treatment. Temperature and pulse rate at this time natural. The wound of soft parts almost healed. No swelling, tenderness or pain about the leg, and patient in good general health.

M. F., æt. 54, a butcher, jumped to the ground from a moving wagon, and was admitted to the Hospital September 15th with a compound fracture of the tibia and fibula of right leg at a little below their middle, the tibia being comminuted. The laceration of soft parts was on anterior aspect of leg, three-fourths of an inch long; "posterior plaster splint," applied with a wood-wool dressing of soft parts, observing the usual antiseptic precautions. On the eleventh day, the dressings were changed when the wound of soft parts seemed healed, all swelling had subsided and the fracture was then practically simple. Patient discharged on the thirty-third day with a sound limb.

J. A., æt. 29, a carpenter, admitted September 30th, fell about twenty feet, sustaining a compound fracture of right leg; both bones broken with wound of the soft parts on the posterior surface, extending transversely, and about three-fourths of an inch in length. The roller plaster bandage of crinoline was used in this case, applied from the toes to the knee; a fenestra was left opposite and corresponding to the wound described, which was dressed, after thorough irrigation, in the customary way as already explained. This patient is still an inmate of the Hospital (October 25th). The wound of the soft parts has healed, and there appears already to be some union of the fracture. He is free from pain, and general condition is good.

It is hoped that these cases of compound fracture may prove of interest, as showing the good results that may be obtained by the immediate treatment of such injuries by plaster of Paris splints or bandage, and particularly as they may serve to draw attention to the advantages that the "posterior plaster splint" presents for fractures of the leg.

